# Characterization of the complete plastome of *Saposhnikovia divaricata* (Turcz.) Schischk

**DOI:** 10.1080/23802359.2020.1715891

**Published:** 2020-01-29

**Authors:** Lingli Li, Maolin Geng, Yingsuo Li, Zenglai Xu, Ming Xu, Mimi Li

**Affiliations:** a Institute of Botany, Jiangsu Province and Chinese Academy of Sciences, Nanjing, China;; b The Jiangsu Provincial Platform for Conservation and Utilization of Agricultural Germplasm, Nanjing, China

**Keywords:** *Saposhnikovia divaricata*, Apiaceae, plastome, chloroplast genome

## Abstract

*Saposhnikovia divaricata* is traditional herbal medicine with a long history in China. We reported the complete chloroplast genome of *S. divaricate* using the next generation sequencing. A total of 115 unique genes were annotated, consisting of 81 protein coding genes, 30 tRNA and 4 rRNA. The overall AT content was 69.2%. The molecular phylogenetic tree reveals that *S. divaricate* is closely related to *Peucedanum* in tribe Selineae.

*Saposhnikovia divaricata* (Turcz.) Schischk is a traditional Chinese medicinal herb belong to Apiaceae (Umbelliferae). The dried roots of *S. divaricate*, commonly called “Fangfeng” or Saposhnikoviae Radix in China, are wildly used for treatment of headache, rheumatoid arthritis and tetanus (National Pharmacopoeia Committee 2015). Several species of family Apiaceae, *Seseli mairei*, *S. yunnanense*, *Pimpinella candolleana* and *Leucas ciliate*, with the similar common name of *S. divaricate*, are misused in traditional Chinese medicine markets (Li et al. 2013). In this study, We assembled and annotated the complete chloroplast genome of *S. divaricate* to authenticate species of *S. divaricata*, and to illustrate phylogenetic inferences of Apiaceae.

The leaf materials of *S. divaricata* were collected from Nanjing Botanical Garden MEM. Sun Yat-Sen, China (32°3′22″N, 118°49′42″E). The specimen was preserved in the Herbarium of Institute of Botany, Jiangsu Province and Chinese Academy of Sciences (NAS) under accession number 0609394. Total genomic DNA of *S. divaricata* was extracted using CTAB method (Doyle and Doyle 1987) and sequenced by Illumina Hiseq X-ten platform (San Diego, CA). The raw reads were assembled by Novoplasty 2.7.2 (Dierckxsens et al. 2017).

The complete plastome sequence of *S. divaricata* (GenBank Accession Number: MN857472) was a double-strand circular DNA molecule of 147,834 bp, with a large single copy (LSC) of 93,202 bp, a small single copy (SSC) of 17,324 bp, and two inverted repeats (IRs) of 18,654 bp. The overall AT content of *S. divaricate* was 69.2%. The chloroplast genome harbored 115 distinct genes, including 81 protein-coding genes (CDS), 30 transfer RNA genes (tRNA), and 4 ribosomal RNA genes (rRNA). Sixteen genes, including *rps*12, contained a single intron, whereas *clp*P and *ycf*3 had two introns.

In order to reconstruct the phylogenetic relationships within Apiaceae, the software PhyloSuite 1.1.16 (Zhang et al. 2020) were employed to obtain 41 additional complete chloroplast genomes of Apiaceae and three of Araliaceae from Genbank. All chloroplast genomes were aligned using the MAFFT 7.409 (Katoh and Standley 2013). And the phylogenetic inference was generated based on maximum likelihood (ML) method in RAxML 8.0 (Stamatakis 2014). The plastome phylogenomic tree reveals that *S. divaricata* is sister to the species from genus *Peucedanum* in tribe Selineae (Figure 1).

**Figure 1.**
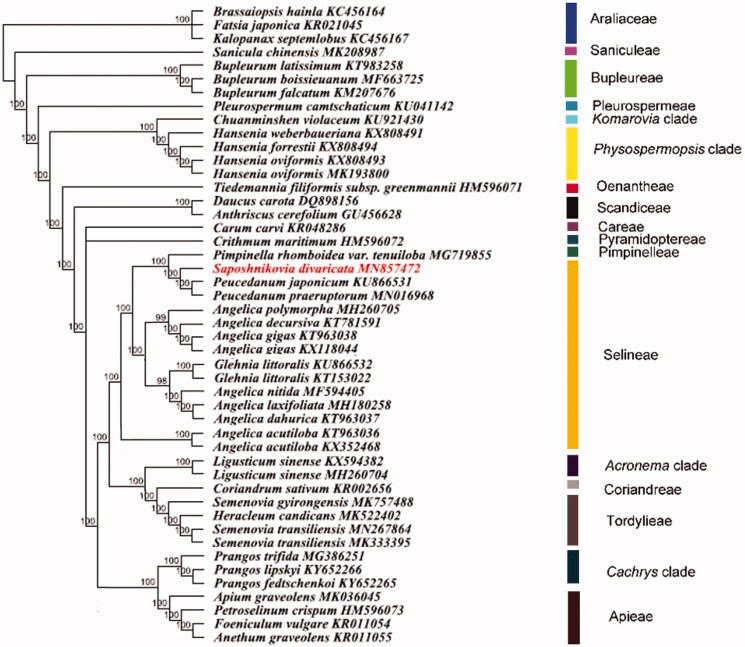
The plastome phylogenomic relationships of Apiaceae based on maximum likelihood method using Araliaceae as outgroup. The number above branches indicated the bootstrap support value from 1000 replicates.
